# Use of Virtual Reality in School-Aged Children with Developmental Coordination Disorder: A Novel Approach

**DOI:** 10.3390/s24175578

**Published:** 2024-08-28

**Authors:** Giulia Purpura, Valentina Di Giusto, Carla Fulvia Zorzi, Giusi Figliano, Mattia Randazzo, Valentina Volpicelli, Rosanna Blonda, Elena Brazzoli, Tarjn Reina, Silvia Rezzonico, Roberta Sala, Anna Cavallini

**Affiliations:** 1 School of Medicine and Surgery, University of Milano Bicocca, 20900 Monza, Italy; giulia.purpura@unimib.it; 2 IRCCS Fondazione Don Carlo Gnocchi, 20148 Milan, Italy; vdigiusto@dongnocchi.it (V.D.G.); czorzi@dongnocchi.it (C.F.Z.); or giusi.figliano1@unicatt.it (G.F.); mrandazzo@dongnocchi.it (M.R.); rblonda@dongnocchi.it (R.B.); ebrazzoli@dongnocchi.it (E.B.); treina@dongnocchi.it (T.R.); srezzonico@dongnocchi.it (S.R.); rsala@dongnocchi.it (R.S.); 3 Research Unit on Theory of Mind, Department of Psychology, Università Cattolica del Sacro Cuore, 20123 Milan, Italy

**Keywords:** developmental coordination disorder, neurodevelopmental disorders, virtual reality, technology, rehabilitation, children

## Abstract

Virtual reality (VR) applications in paediatric rehabilitation are recent but promising. This brief report describes a VR rehabilitation program for a small sample of children with Developmental Coordination Disorder (DCD). The program focused explicitly on executive functions, a key area of concern for this population. It was conducted over 11 weeks in the CARE Lab. This lab was designed with appropriate structural characteristics and sophisticated technology to provide a rehabilitative setting with recreational and semi-immersive features. Before and after the VR training, the children were evaluated in terms of visual attention, inhibition, planning abilities, and visual–motor coordination. The rehabilitation programs were customised according to the clinical needs and the functional profile of each patient, proposing different games with variable complexity levels. These preliminary results showed a global and clinically significant change in executive functions, especially visual attention and inhibition skills. These findings suggest interesting implications for clinical practice, providing new information for professionals regarding the application of VR in the field of paediatric rehabilitation.

## 1. Introduction

Developmental Coordination Disorder (DCD) is a neurodevelopmental motor disorder characterised by marked impairment in motor coordination and the performance of motor activities (e.g., walking and handwriting), being well below the expectation for the child’s chronological age but not related to medical conditions such as cerebral palsy, muscular dystrophy, visual impairment, or intellectual disability [1]. During preschool and school age, children with DCD are usually considered “clumsy” and show specific impairment in gross-motor and fine-motor coordination, which impacts their school performance and may restrict participation [2]. Among these, difficulties in balance and postural control [3,4], as well as in bimanual and unimanual abilities, are widely described, and, in particular, impairment in the efficient use of the non-dominant hand was frequently found [5,6,7]. Moreover, several authors reported atypical learning deficits in visuomotor adaptation and motor sequence learning tasks, and it was suggested that this poor motor performance in DCD children may be attributed to disrupted brain function [8]. Although the clinical manifestations of this neurodevelopmental disorder are more evident from 4–5 years of age, these children may show, during early development, delays in some milestones that do not involve the motor domains exclusively, such as walking, but also some neuropsychological functions, for example, in maintaining attention during daily routines [9]. In fact, despite the constancy of the motor core symptoms, heterogeneity exists in the nature and severity of the motor impairment and the sensory and cognitive problems often associated with DCD. Lust and colleagues [10] identified four different clusters of DCD functioning based on performance profiles across measures of perceptual–motor, cognitive, and other neuropsychological functions. Van Dyck et al. [11] reported that reduced executive functions were present across the clusters of children with DCD. Similarly, Sartori and collaborators [12,13] confirmed that children with DCD showed significantly lower scores than children with typical development on all the measures of working memory, inhibitory control, and cognitive flexibility. The same authors also highlighted that lower executive functions, particularly inhibition and visuospatial memory, predicted lower school performance in these children. This aligns with data from Querne et al. [14] that, using a connectivity study, suggested a less efficient engagement of the inhibition cerebral network in children with DCD than in the controls. However, a specific automatisation deficit linked to cerebellar dysfunction in this population was also hypothesised by several authors [15].

So, due to the complexity of this neurodevelopmental disorder, according to the guidelines provided by the European Academy of Childhood Disability (EACD), the diagnosis of DCD requires a global assessment by a multidisciplinary team of experts [16]. This team should be capable of evaluating the strong and weak points of the child using standardised assessment tools for planning adequate and evidence-based rehabilitation programs that consider the entire profile of functioning. According to the same document, it is possible to group interventions for children with DCD into three categories: (1) body function and structure-oriented, where the activity is designed to improve specific body functions considered to underlie the functional motor deficit; (2) activity-oriented, where the activity is designed to improve the performance in specific tasks; and (3) participation-oriented, where the activity is designed to improve participation during everyday life activities. In particular, the activity-oriented or participation-oriented approaches seem to be more efficient, yielding better functional performance outcomes in less time for children with DCD [16]. For this reason, several authors [12,17,18] emphasised the need for task-specific intervention programs that implement executive functions to promote the learning mechanisms in this population, especially in subgroups with persistent motor coordination problems during late school age. This aspect is essential since EFs are critical for success in all aspects of life and are sometimes more predictive than IQ or socioeconomic status [19].

In this context, immersive technologies such as virtual reality (VR) were recently applied in paediatric rehabilitation. Nevertheless, a recent review by Lino et al. [20] underlines that only a few studies explore the use of VR with DCD children and that, in these studies, the authors often adapt off-the-shelf tools (such as Nintendo Wii Fit and Sony’s PlayStation Eye Toy) that are not specifically designed for rehabilitation. While these tools are cost-effective and easy to use, they do not enable customising the tasks based on the specific motor and neuropsychological deficits. Indeed, for its intrinsic characteristics, VR may help professionals to improve children’s ability to manipulate mental images, including the ability to rotate objects mentally, visualise spaces, orient themselves in space, and program movements, which were demonstrated to be impaired in children with DCD [21]. Moreover, according to some authors, VR use is a significant motivational factor as the child is immersed in a more natural environment, and the feedback provided by play is prompt during activities [22]. Also, the high number of movement repetitions achieved through VR practice [23] and the resulting high treatment adherence [24] are key factors to consider in improving the effectiveness of such rehabilitation protocols.

Because of the critical role of EFs in regulating, monitoring, and controlling motor behaviour toward a goal, we present the results of a new VR-based rehabilitation program in a small group of children with DCD. This VR program has already been applied to children with Specified Learning Disorders [25], demonstrating itself as feasible and helpful for this specific aim. Specifically, the VR intervention was performed in the context of the CARE-Lab. The CARE-Lab is a space designed for engaging and technology-based rehabilitation. It is powered by VITAMIN, a Medical Device software that enables the integration of different sensors (e.g., Microsoft Kinect V2 and Wii Balance Board) to run a variety of exergames. A multidisciplinary team developed the VITAMIN digital platform to customise the approach based on each child’s functional profile. These preliminary data can provide interesting insights for the development of randomised controlled trials to assess the efficacy of this novel method in improving the neuropsychological and motor skills of children with DCD.

## 2. Materials and Methods

### 2.1. Participants

Our study involved ten school-aged children (6M, 4 F; age range: 7–9 years) with a diagnosis of DCD, assessed by a multidisciplinary team according to DSM-5 criteria [1]. Children were recruited from the Division of Child and Adolescent Neuropsychiatry of the IRCCS Don Gnocchi Foundation—Santa Maria Nascente of Milan (Italy). Parental/legal guardian consent was requested to include children in this study. Recruitment was carried out using convenience sampling and in accordance with the principles of the Declaration of Helsinki. The local Research Ethics Committee approved all procedures involving human subjects in this study (08_13/10/2020 Ethics Committee Fondazione Don Carlo Gnocchi). Characteristics of the sample are reported in Table 1.

### 2.2. Outcome Measures

Clinical outcome measures

Before and after training, the neuropsychiatry team used a specific clinical protocol to assess three principal EFs (visual attention, inhibition, and planning).

(a) “Visual Attention Subtest” of the Italian Version of Developmental Neuropsychological Assessment—Second Edition (NEPSY-II) was used to assess visual attention skills [26]. This subtest is a time trial of barrage that evaluates how well children can focus on visual target stimuli amidst distractor stimuli: it can be considered a dual-tasking test as subjects are required to split their attention to different elements simultaneously. The Scaled Score, standardised for age, from the test manual (range of 0–19, average score of 10) was obtained by converting the raw score (the number of correct answers minus errors).
(b) To evaluate inhibition skills, the “Inhibition Subtest” from NEPSY-II [26] was administered. In this subtest, the child is asked to identify black-and-white shapes or arrows and name the shape, direction, or an alternate response based on the colour of the shape or arrow. Part A (Naming Condition) and Part B (Inhibition Condition) of the test were used for this study. The Naming Condition requires participants to name the shape of squares and circles or the up or down direction of arrows, so it evaluates, in particular, the capacity to assign the correct name to a figure rapidly; the Inhibition Condition requires participants to provide the opposite naming response on the same stimuli (e.g., if the child is shown a circle, s/he must say “square”; if s/he sees a square, s/he must say “circle”). Performance requires focused attention, verbal memory, resistance to interference, and rapid automatised naming. The Scaled Scores, standardised for the age, from the test manual (range of 0–19, average score of 10) were obtained based on time.
(c) “Mazes Subtest” of the WISC-III [27] was included to evaluate planning abilities. It consists of 10 mazes of various sizes and complexity. The objective is for the child to draw a line from the centre to the outside of each maze without intersecting any lines representing walls. All items are timed. Based on age, the Standard Score is obtained from the test’s manual (range: 3–19) through a ratio between execution time and the number of errors allowed (range raw scores: 0–28).

Technological outcome measures

Before and after training, a specific task using the VR technology of the CARE Lab was also administered to assess each child’s performance in terms of accuracy and area of visual–motor coordination and motor exploration in a virtual reality context. During this task, similar to the game “Gita al Parco” (see Section 2.3) that is used during the intervention, several moving targets and distractors appear on the screen. The child must reach them with the upper limb or avoid them. Every task is performed separately with the dominant and non-dominant limbs to verify their efficiency. In this way, aside from clinical scales, we can exploit the quantitative data recorded by the sensors. These data allowed us to evaluate the children’s performances through objective and quantitative measures as well. In particular, we focused on the following values:

(a) Percentage of bonus targets (***B***_%_): it is evaluated as the ratio of bonus targets that the children hit (***B_hit_***) by the total number of bonus targets spawned (***B_spawned_***). A higher percentage of bonus hit is indicative of a better performance.
(b) Percentage of malus targets (***M***_%_): it is evaluated as the ratio of malus targets that the children hit (***M_hit_***) by the total number of malus targets spawned (***M_spawned_***). A better performance is indicated by a lower percentage of malus targets hit.
(c) Percentage of screen area the children cover during the task (***A***_%_): it is estimated using the concave hull method. The concave hull is an evolution of the convex hull, representing the smallest convex polygon containing all the input points. While only one convex hull exists per cloud of point, this is not generally true for the concave hull, as more than one solution can be accepted depending on the final application (for more details, see Ref. [28]). In our case, we estimated the concave hull points following the k-nearest method proposed by Moreira and Santos [28]. Given this set of points, we estimated the area of the hull with the *Shoelace formula*, also called Gauss’s area formula or Suveryor’s Area formula (***A_shoelace_***) [29]. Finally, we divided this area by the total screen area (***A_screen_***).

### 2.3. Computer-Assisted Rehabilitation Laboratory (CARE Lab)

The CARE Lab (Computer Assisted REhabilitation Laboratory), which is located at the IRCCS Don Gnocchi Foundation in Milan, is a physical space for studying and integrating innovative and high-tech solutions and their effectiveness in clinical practice through a multidisciplinary approach, specifically for the paediatric population. For this purpose, the CARE Lab has sensors that provide continuous feedback to the child and record patient movement data during rehabilitation sessions (i.e., Kinect—Microsoft, Redmond, WA, USA, and Wii Balance Board—Nintendo, Kyoto, Japan). The setting consists of two separate rooms to guarantee the acceptableness of the rehabilitation intervention. The “high-tech” room, where the intervention takes place, was developed with the aim of reducing its artificiality aspect as much as possible. Thus, side walls are covered with colourful cartoonlike playground scenes to hide the sophisticated technology so the child can experience a rehabilitative setting with recreational and semi-immersive features (i.e., screen, sound appliances, and projectors). This was an essential point of the project because it promoted the child’s adaptation to the rehabilitation context [25,30,31]. In this room, a frontal white screen covers almost the entire field of view of the child looking forward, and it is used to project the games of the rehabilitation sessions; in the rear wall, a two-way mirror is hidden by the picture of an ice cream van; on the floor, it is possible to project images and videos. The front screen projector has high-resolution (full HD) and lumen capabilities to ensure sharp and bright images. The room has been acoustically equipped in order to minimise reverberations, and it is also possible to adjust the lighting (either in terms of intensity or colour). The “control room” houses the core multimedia and computing capabilities of the CARE Lab (for more details, see Refs. [30,31]).

In the context of the CARE Lab, a software architecture called VITAMIN (VIrtual realiTy plAtform for Motor and cognItive rehabilitatioN) was developed and implemented. VITAMIN is a medical-grade software designed and built by Don Carlo Gnocchi Foundation. It integrates sensors, runs exergames, and stores data with three software modules (Sensor Interface, Control Hub, and Game Engine) developed in C#.NET, Python, and 3D graphical engines (Unreal Engine and Unity 3D) for the three modules, respectively. Through this software, the child’s movements, acquired by sensing devices, are translated into digital information. For example, the continuous recording of the virtual hand’s position during each game allows for the reconstruction of the whole trajectory and the derivation of specific quantitative indexes (for more details, see Refs. [30,31]). At the beginning of the session, a calibration procedure of around three minutes must be performed. The rehabilitation programs were customised according to clinical needs and the characteristics of each child, offering different games with varying levels of complexity.

**Figure 1:**
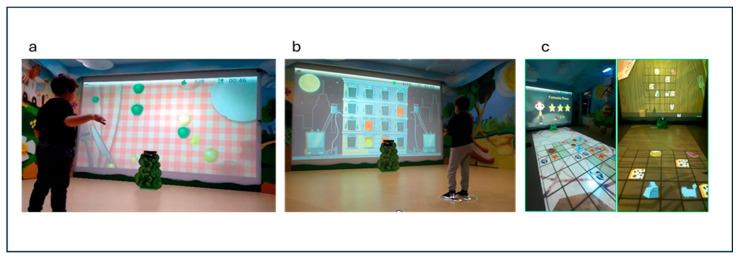
Examples of game activity: (**a**) Gita al Parco; (**b**) Pronti Via!; (**c**) Passo, passo. Images of games on VITAMIN reproduced with permission from IRCCS Fondazione Don Gnocchi of Milan (Italy).

There are three available games on VITAMIN (see Figure 1) as follows:

(a) “Gita al parco” (Italian for “Trip to the park”): This game offers moving targets and distractors on the screen that the subject must reach with the upper limb or avoid (for example, to reach the large green and small yellow apples and to avoid the large yellow apples). This game focuses on visual attention, inhibitory motor control, shifting, and upper limb quality movements (adduction–abduction in the frontal plane). A constant ability to plan and control the motor gesture is required since the targets appear randomly and are in continuous movement.
(b) “Pronti, via!” (Italian for “Ready, go!”): In this game, a mixed sequence of targets (from a minimum of 2 to a maximum of 5), with some distractor (from a minimum of 0 to a maximum of 2) is proposed to the child on the screen. Children must memorise the sequence and hit the targets (for example, three princesses that appear in a specific spatial sequence on the screen) by a specific flexion and extension arm’s movements to reproduce the sequence in exact or reverse order (to exercise sequential and visuospatial memory). This game focuses on EFs like visual attention, working memory, and upper limb control movements.
(c) “Passo, passo” (Italian for “Step by step”): during this game, the child is engaged in an activity in which he is moving within a grid projected on the floor. Its goal is to reach specific targets within the grid, following a path that is as direct and precise as possible. Along the way, the child must be careful to prevent unnecessary detours and maintain focus on the final goal. The grid consists of a series of boxes, some of which are active while others are passive. The active boxes present challenges, such as mini-games that require the child to complete exercises such as balancing on one leg, shifting body weight from side to side, or performing light squats, while other active boxes must be avoided because they include distractor targets that, if stepped on, force the child to re-start the game and re-plan the most suitable path. Passive boxes, on the other hand, do not contain additional challenges or exercises. These boxes serve as transit points, allowing the child to continue toward the target without interruption. This activity helps the child to develop motor, problem-solving, and planning skills.

Intervention procedures

For this project, children participated in eleven weeks of individualised VR training in the CARE Lab (two weekly sessions of 45 min). All three available games on VITAMIN (see Figure 1) were used. During each session, some short breaks were included based on subject performance and tiredness. The therapist monitored and, when appropriate, modified the levels of complexity of the exercises at each session. In tasks that provide unimanual movements, therapists can decide when to exercise the non-dominant or dominant hand.

### 2.4. Statistical Analysis

Statistical analyses were carried out using JASP (Version 0.17.2) software. A *p*-value below 0.05 was interpreted as significant. Descriptive analyses were reported where appropriate: the continuous variables were expressed as the mean ± standard deviation of the corresponding distribution, and the mean percentage of amelioration was reported for each measure following the formula

$$∆=\frac{T1-T0}{T0}\;*\;100$$

Since the sample size was limited, we used a non-parametric approach to statistically analyse the children’s scores. The Wilcoxon Test was used to determine whether there were significant differences in different parameters across the two time points (before and after training). For the Wilcoxon Test, effect size was obtained by the matched rank biserial correlation (small effect, r = 0.2; medium effect, r = 0.5; large effect, r ≥ 0.8). Regarding the technological outcome measures, every index was separately analysed for the dominant and non-dominant hand.

## 3. Results

**Figure 2:**
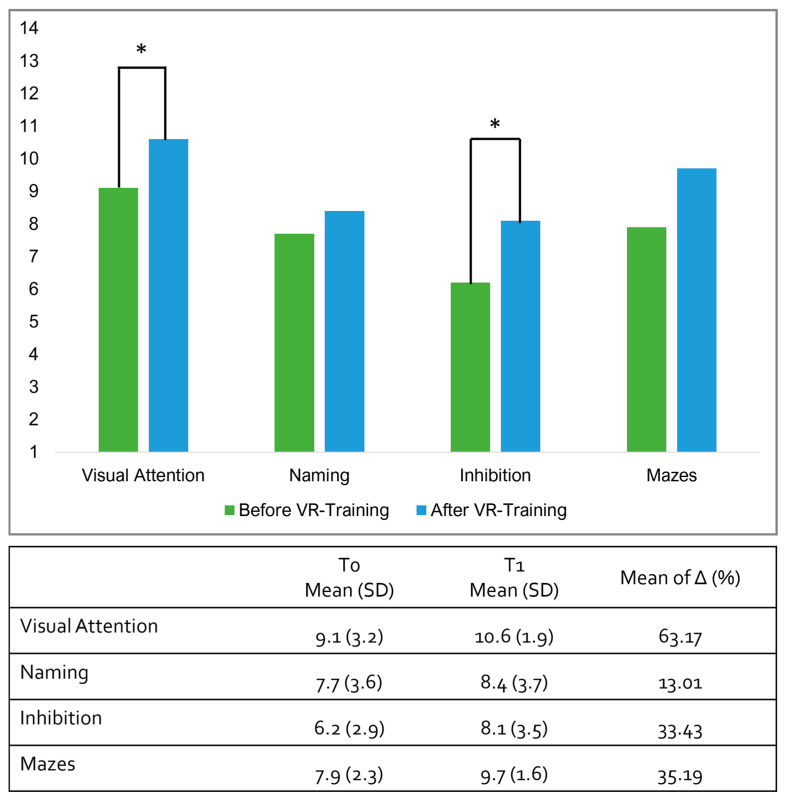
Graphic representation of mean scores in the four clinical outcome measures before and after VR training. The asterisks indicate significant differences.

All the participants completed 100% of the training sessions. As regards the clinical outcome measures, between the pre-training evaluation and the post-training evaluation, the Wilcoxon Test showed a statistical difference in the Visual Attention Subtest (pre-training: mean 9.1, SD: 3.2; post-training: mean 10.6, SD 1.9; *p* = 0.049, effect size r = 0.857) and in Part B of “Inhibition Subtest” (pre-training: mean 6.2, SD: 2.9; post-training: mean 8.1, SD 3.5 *p* = 0.021, effect size r = 1.000). Moreover, a tendency to significance was also evident in the comparison of the scores regarding the “Mazes Subtest” between the pre-training evaluation and post-training evaluation (pre-training: mean 7.9, SD: 2.3; post-training: mean 9.7, SD 1.6 *p* = 0.073, effect size r = 0.689). No significant difference was found in Part A (Naming Condition) of the “Inhibition Subtest” (pre-training: mean 7.7, SD: 3.6; post-training: mean 8.4, SD 3.7 *p* = 0.262, effect size r = 0.500). More details are reported in Figure 2.

As regards the technological outcome measures, between the pre-training evaluation and the post-training evaluation, the Wilcoxon Test showed a statistical difference in the percentage of bonuses hit, which was increased, confirming a performance improvement, both with the dominant hand and with the non-dominant hand (dominant hand: *p* = 0.010; effect size r = 0.891; non-dominant hand: *p* = 0.006, effect size r = 1.000). Moreover, a statistically significant decrease in the percentage of malus targets hit with both hands, which also confirms the performance improvement, was also revealed (dominant hand: *p* = 0.014, effect size r = 1.000; non-dominant hand: *p* = 0.020, effect size r = 0.818). No significant difference was found in the percentages of covered area (dominant hand: *p* = 0.333; effect size r = 0.364; non-dominant hand: *p* = 1.000, effect size r = 0.018). More details are reported in Table 2.

## 4. Discussion

The main focus of this study was to explore the feasibility of an eleven-week VR intervention in children with DCD. We wanted to investigate whether a VR-based program could improve the functioning of this population during the school age. Despite the small sample size, statistically significant changes were noted in some of the outcome measures, with medium to large effect sizes. We have chosen to investigate the impact of this method on EFs because, in children with DCD, motor difficulties are closely associated with lower abilities in some neuropsychological domains related to planning, controlling, and organising goal-directed behaviours. This is supported by data from Serdarevic and collaborators [32], who found that less optimal neuromotor development in the early stages of life is predictive of lower performance on visuospatial, immediate visual memory, shifting, and planning tasks at later ages. Also, Asonitou et al. [33,34] reported a strong relationship among perception, visuospatial working memory, and visuomotor coordination. These authors also underlined that the simultaneous and attentional processes are often poorer in children with DCD than in children without DCD. As a matter of fact, Roebers and Kauer [35] suggested that motor skills and EFs share the same underlying processes, including processing information, organisation of behaviour, attention to the task, and inhibition of irrelevant stimuli. Moreover, combined motor difficulties and problems in other non-motor areas add to the complexities of understanding the causes and predicting the development of DCD. 

In fact, it is widely recognised that many children with this diagnosis also manifest attention and concentration problems or other learning disorders [15]. Several studies have highlighted that Attention Deficit/Hyperactivity Disorder, reading difficulties, Specific Learning Disorders, and Specific Language Disorders are frequently associated with symptoms of DCD [36,37,38]. A recent review of the literature [39] points to the emerging evidence of the other comorbidities related to DCD, including autism, non-verbal learning disorder, intellectual disability, tic disorder, and others. Thus, the comorbidities associated with DCD further suggest the complexity and heterogeneity of this neurodevelopmental disorder. This confirms the importance of a multidisciplinary diagnosis to identify the different impairment areas to form the foundation of an effective rehabilitation treatment.

In line with these findings, the results of this brief report support the feasibility of using VR-based interventions with DCD children to improve their EFs. Although the absence of a control group and the scarceness of the sample make these results only preliminary, the clinical outcome measures and technological outcome measures collected by the CARE Lab suggest that an activity-oriented program based on a VR approach may enhance visual attention, inhibition, and specific visual–motor coordination abilities. In addition, regarding planning and naming abilities, although the statistical analyses may not show significance, the observed increase in the percentage of amelioration suggests a clinical change. The lower improvement in the naming test may be because this task focuses mainly on language speed, which was not the primary goal of this study’s rehabilitation program. Obviously, several biases and limitations do not permit us to provide conclusions about the effectiveness of this approach. Still, it is also true that the preliminary data suggest several considerations about the application of VR in the rehabilitation of children with DCD. This innovative approach might be used to exercise motor abilities and, above all, to organise better cognitive strategies for motor problem-solving.

Based on these issues, the combined technologies of the CARE Lab and VITAMIN might be helpful in rehabilitating the EFs and visual–motor coordination in children with DCD. However, this brief report does not analyse some motor control strategies of children, including the hip and ankle strategies for balance control or the centre of pressure [40]. In the future, these analyses could also be integrated.

Another important point that supports our data is that the intervention was well accepted by the children, who showed high motivation during the entire training period.

The gamification of therapy, also by using the new high-tech tools available in our society, can bring in fun and could thus make therapeutic interventions more meaningful [41]. This is essential in facilitating positive changes in children with different types of neurodevelopmental disorders. Although other authors have already investigated the use of VR for rehabilitating children with DCD [42,43,44,45], most of these studies focused on motor abilities without considering the higher cognitive processes necessary to carry out complex and finalised actions. By contrast, our study aligns with the results of EbrahimiSani et al. [46], which demonstrated the effectiveness of VR training on the feedforward motor control functions of DCD children.

In conclusion, VR rehabilitation is promising when used with traditional rehabilitation programs. This is because it could offer active learning in DCD children, focusing on the underlying cognitive processes involved in motor control. These preliminary results are encouraging and support the importance of developing and implementing randomised controlled trials to better investigate this approach’s effects on children’s participation.

## Figures and Tables

**Table 1:** Principal characteristics of participants.

	Gender	Age	Dominant Hand *	IQ	Comorbidities **
**S1**	M	7	R	106	DG
**S2**	M	8	R	102	DG
**S3**	M	9	R	90	DG + DC
**S4**	M	8	L	101	DG
**S5**	M	9	R	71	DL + DO + DG + DC
**S6**	F	7	R	83	DL + DG
**S7**	F	9	R	102	DG + DO
**S8**	F	7	L	92	DL + DO + DG
**S9**	F	8	R	100	DG + DO
**S10**	M	7	R	82	DG

Abbreviations: * R, right, L, left; ** DL, dyslexia; DO, dysorthography; DG, dysgraphia; DC, dyscalculia.

**Table 2:** Results from technological outcome measures before and after training. * *p* ≤ 0.05, ** *p* ≤ 0.01.

Indexes	Pre-Training *Mean (SD)*	Post-Training *Mean (SD)*	*p*-Value *(Wilcoxon Test)*	Effect Size	Mean of Δelta *(%of Amelioration)*
Bonus (%)—Dominant Hand	70.42 (13.5)	84.53(12.1)	0.010 *	large	21.7
Malus (%)—Dominant Hand	26.16 (16.8)	10.0 (8.4)	0.014 *	large	−45.8
Covered Area (%)—Dominant Hand	65.89 (5.5)	62.0 (7.9)	0.333	small	−4.6
Bonus (%)—Non-Dominant Hand	67.01 (13.8)	83.85 (10.5)	0.006 **	large	28.5
Malus (%)—Non-Dominant Hand	23.45 (12.5)	14.8 (11.6)	0.020 *	large	−36.4
Covered Area (%)—Non-Dominant Hand	65.50 (9.7)	65.69 (8.5)	1.000	null	2.1

## Data Availability

The data analysed in this study are subject to the following licenses/restrictions: the data are not publicly available for ethical reasons. The data that support this study’s findings are available from the corresponding author upon request. Requests to access these datasets should be directed to A.C., ancavallini@dongnocchi.it.

## References

[1] APA—American Psychiatric Association (2013). Diagnostic and Statistical Manual of Mental Disorders.

[2] Biotteau M., Albaret J.M., Chaix Y. (2020). Chapter 1—Developmental coordination disorder. Handbook of Clinical Neurology.

[3] Geuze R.H. (2005). Postural Control in Children with Developmental Coordination Disorder. Neural Plast..

[4] Johnston L.M., Burns Y.R., Brauer S.G., A Richardson C. (2002). Differences in postural control and movement performance during goal directed reaching in children with developmental coordination disorder. Hum. Mov. Sci..

[5] Sigmundsson H., Whiting H. (2002). Hand Preference in Children with Developmental Coordination Disorders: Cause and Effect?. Brain Cogn..

[6] Sigmundsson H., Ingvaldsen R.P., Whiting H.T.A. (1997). Inter- and intra-sensory modality matching in children with hand-eye co-ordination problems. Exp. Brain Res..

[7] Grohs M.N., Hawe R.L., Dukelow S.P., Dewey D. (2021). Unimanual and bimanual motor performance in children with developmental coordination disorder (DCD) provide evidence for underlying motor control deficits. Sci. Rep..

[8] Bo J., Lee C.-M. (2013). Motor skill learning in children with Developmental Coordination Disorder. Res. Dev. Disabil..

[9] López A.G., Madrid V.C., Hidalgo-Robles Á., Gutiérrez-Ortega M. (2022). Early signs of functioning and contextual factors in children 0 to 6 years of age at high risk of or with developmental coordination disorder: A scoping review. Child Care Health Dev..

[10] Lust J.M., Steenbergen B., Diepstraten J.E.M., Wilson P.H., Schoemaker M.M., Poelma M.J. (2022). The subtypes of developmental coordination disorder. Dev. Med. Child Neurol..

[11] Van Dyck D., Baijot S., Aeby A., De Tiège X., Deconinck N. (2022). Cognitive, perceptual, and motor profiles of school-aged children with developmental coordination disorder. Front. Psychol..

[12] Sartori R.F., Valentini N.C., Fonseca R.P. (2019). A comparative study of executive function in children with and without developmental coordination disorder. Child Care Health Dev..

[13] Sartori R.F., Nobre G.C., Fonseca R.P., Valentini N.C. (2021). Do executive functions and gross motor skills predict writing and mathematical performance in children with developmental coordination disorder?. Appl. Neuropsychol. Child.

[14] Querne L., Berquin P., Vernier-Hauvette M.-P., Fall S., Deltour L., Meyer M.-E., de Marco G. (2008). Dysfunction of the attentional brain network in children with Developmental Coordination Disorder: A fMRI study. Brain Res..

[15] Visser J. (2003). Developmental coordination disorder: A review of research on subtypes and comorbidities. Hum. Mov. Sci..

[16] Blank R., Barnett A.L., Cairney J., Green D., Kirby A., Polatajko H., Rosenblum S., Smits-Engelsman B., Sugden D., Wilson P. (2019). International clinical practice recommendations on the definition, diagnosis, assessment, intervention, and psychosocial aspects of developmental coordination disorder. Dev. Med. Child Neurol..

[17] Michel E., Molitor S., Schneider W. (2016). Differential changes in the development of motor coordination and executive functions in children with motor coordination impairments. Child Neuropsychol..

[18] Omer S., Leonard H.C. (2020). Internalising symptoms in Developmental Coordination Disorder: The indirect effect of everyday executive function. Res. Dev. Disabil..

[19] Diamond A., Ling D.S. (2016). Conclusions about interventions, programs, and approaches for improving executive functions that appear justified and those that, despite much hype, do not. Dev. Cogn. Neurosci..

[20] Lino F., Arcangeli V., Chieffo D.P.R. (2021). The Virtual Challenge: Virtual Reality Tools for Intervention in Children with Developmental Coordination Disorder. Children.

[21] Adams I.L., Lust J.M., Wilson P.H., Steenbergen B. (2014). Compromised motor control in children with DCD: A deficit in the internal model?—A systematic review. Neurosci. Biobehav. Rev..

[22] Wang M., Reid D. (2010). Virtual Reality in Pediatric Neurorehabilitation: Attention Deficit Hyperactivity Disorder, Autism and Cerebral Palsy. Neuroepidemiology.

[23] Holden M.K. (2005). Virtual Environments for Motor Rehabilitation: Review. CyberPsychol. Behav..

[24] Gordon C., Roopchand-Martin S., Gregg A. (2012). Potential of the Nintendo Wii™ as a rehabilitation tool for children with cerebral palsy in a developing country: A pilot study. Physiotherapy.

[25] Di Giusto V., Purpura G., Zorzi C.F., Blonda R., Brazzoli E., Meriggi P., Reina T., Rezzonico S., Sala R., Olivieri I. (2023). Virtual reality rehabilitation program on executive functions of children with specific learning disorders: A pilot study. Front. Psychol..

[26] Urgesi C., Campanella F., Fabbro F. (2011). La Versione Italiana Della NEPSY-II per la Valutazione Neuropsicologica ad Ampio Raggio del Bambino da 3 a 16 Anni.

[27] Wechsler D. (1991). Wechsler Intelligence Scale for Children—Third Edition (WISC-III).

[28] Moreira A., Santos M.Y. (2007). Concave Hull: A k-nearest neighbours approach for the computation of the region occupied by a set of points. Proceedings of the 2nd International Conference on Computer Graphics Theory and Applications (VISIGRAPP 2007).

[29] Braden B. (1986). The Surveyor’s Area Formula. Coll. Math. J..

[30] Olivieri I., Meriggi P., Fedeli C., Brazzoli E., Castagna A., Roidi M.L.R., Angelini L. (2018). Computer Assisted REhabilitation (CARE) Lab: A novel approach towards Pediatric Rehabilitation 2.0. J. Pediatr. Rehabil. Med..

[31] Meriggi P., Mandalà M., Randazzo M., Brazzoli E., Castagna A., Di Giusto V., Cavallini A., Marzegan A., Lencioni T., Olivieri I. (2024). Non-immersive virtual reality based treatment for children with unilateral cerebral palsy: Preliminary results. J. Pediatr. Rehabil. Med..

[32] Serdarevic F., van Batenburg-Eddes T., Mous S.E., White T., Hofman A., Jaddoe V.W., Verhulst F.C., Ghassabian A., Tiemeier H. (2015). Relation of infant motor development with nonverbal intelligence, language comprehension and neuropsychological functioning in childhood: A population-based study. Dev. Sci..

[33] Asonitou K., Koutsouki D., Kourtessis T., Charitou S. (2012). Motor and cognitive performance differences between children with and without developmental coordination disorder (DCD). Res. Dev. Disabil..

[34] Asonitou K., Koutsouki D. (2016). Cognitive process-based subtypes of developmental coordination disorder (DCD). Hum. Mov. Sci..

[35] Roebers C.M., Kauer M. (2008). Motor and cognitive control in a normative sample of 7-year-olds. Dev. Sci..

[36] Dewey D., Kaplan B.J., Crawford S.G., Wilson B.N. (2002). Developmental coordination disorder: Associated problems in attention, learning, and psychosocial adjustment. Hum. Mov. Sci..

[37] Kadesjö B., Gillberg C. (1998). Attention deficits and clumsiness in Swedish 7-year-old children. Dev. Med. Child Neurol..

[38] Gillberg C., Kadesjö B. (2000). Why Bother About Clumsiness? The Implications of Having Developmental Coordination Disorder (DCD). Neural Plast..

[39] Lino F., Chieffo D.P.R. (2022). Developmental Coordination Disorder and Most Prevalent Comorbidities: A Narrative Review. Children.

[40] Martini G., Beani E., Filogna S., Menici V., Cioni G., Battini R., Sgandurra G. (2022). New Technological Approach for the Evaluation of Postural Control Abilities in Children with Developmental Coordination Disorder. Children.

[41] Dan B. (2022). Gamification of therapy: The fun factor in rehabilitation. Dev. Med. Child Neurol..

[42] Ashkenazi T.M., Weiss P.L.P., Orian D., Laufer Y.D. (2013). Low-Cost Virtual Reality Intervention Program for Children with Developmental Coordination Disorder: A pilot feasibility study. Pediatr. Phys. Ther..

[43] Mentiplay B.F., FitzGerald T.L., Clark R.A., Bower K.J., Denehy L., Spittle A.J. (2019). Do video game interventions improve motor outcomes in children with developmental coordination disorder? A systematic review using the ICF framework. BMC Pediatr..

[44] Ferguson G., Jelsma D., Jelsma J., Smits-Engelsman B. (2013). The efficacy of two task-orientated interventions for children with Developmental Coordination Disorder: Neuromotor Task Training and Nintendo Wii Fit training. Res. Dev. Disabil..

[45] Cavalcante Neto J.L.C., Steenbergen B., Wilson P., Zamunér A.R., Tudella E. (2019). Is Wii-based motor training better than task-specific matched training for children with developmental coordination disorder? A randomized controlled trial. Disabil. Rehabil..

[46] EbrahimiSani S., Sohrabi M., Taheri H., Agdasi M.T., Amiri S. (2020). Effects of virtual reality training intervention on predictive motor control of children with DCD—A randomized controlled trial. Res. Dev. Disabil..

